# Mapping Youth Awareness of Sports Betting Advertising During the 2022 FIFA World Cup

**DOI:** 10.1007/s10899-024-10299-2

**Published:** 2024-04-03

**Authors:** Gianluca Di Censo, Paul Delfabbro, Daniel L. King

**Affiliations:** 1 School of Psychology, The University of Adelaide, Adelaide, Australia; 2 College of Education, Psychology & Social Work, Flinders University, Adelaide, Australia

**Keywords:** Young People, Gambling, Marketing, Promotions, Wagering

## Abstract

Young people are known to be highly engaged in sports betting and therefore may be particularly susceptible to the effects of gambling-related advertising. The purpose of the present study was to examine young people’s recall of sports betting advertising during the 2022 FIFA World Cup. The sample consisted of 190 UK residents aged 18–24 who had watched at least one 2022 World Cup match. A cross-sectional survey was conducted to collect data on participants' recall of sports betting advertisements across several media types and for different bets and betting offers, as well as their problem gambling scores. The findings indicated that young people were able to recall a high amount of advertising for various types of bets (95.6%) and betting inducements (89.5%). A high proportion of young people recalled advertising for risky bet types and promotions, such as 64.2% for in-play betting and 68.1% for sign-up offers. Overall, higher-risk gamblers recalled encountering more advertising than lower-risk gamblers. Participants recalled encountering sports betting advertisements on social media the most (10–14 ads per week), then on internet banners and television (5–9 ads per week, respectively). Less than half (46.3%) of respondents were aware of advertising for responsible gambling tools. This study underscores the need for policy measures that limit young people's exposure to gambling advertising, particularly for products that may contribute to gambling-related harm, and that increase the promotion of responsible gambling tools.

## Introduction

Sports betting has grown in popularity, with 8.3% of adults over the age of 18 reporting participation in the past year (Rockloff et al., 2020). Research indicates that sports betting is more prevalent among younger people, particularly males (Browne et al., 2023; Seal et al., 2022). In fact, between 2018 and 2019, a sizable portion of young people aged 18 to 24 reported participating in sports betting, specifically 21.9% of all young people and 31.9% of young men (Rockloff et al., 2020). This demographic has also been found to be at the highest risk for developing gambling-related problems associated with sports betting (Aragay et al., 2021; Hing et al., 2016; Russell et al., 2019). Although sports betting exhibits lower participation rates in comparison to other forms of gambling (e.g., electronic gaming machines, and horse and dog racing) (Armstrong & Carroll, 2017; Browne et al., 2023; Rockloff et al., 2020), its pervasive advertising across various media platforms raises the question of its potential role in exacerbating gambling-related harm (Deans et al., 2016; Delfabbro et al., 2021; National Audit Office, 2020; Thomas et al., 2018). This is especially pertinent in the context of sports betting advertising that is aired during programming aimed at younger audiences.

Scholarly articles have provided insight into the advertising channels used to market sports betting to young people. Research has shown that younger people frequently encounter sports betting advertisements in a variety of public settings, including sporting events, billboards, bars, and convenience stores (Deans et al., 2017; Pitt et al., 2016; Smith et al., 2019). Gen Z is of particular interest, as it represents a generation that has grown up with social media and is highly engaged with social media platforms such as YouTube, Instagram, Facebook, and Twitch (Auxier & Anderson, 2021; Twenge et al., 2019). Sports betting companies are known to employ social media advertising extensively (Torrance et al., 2021), with the National Audit Office (2020) estimating that it constitutes a substantial portion of their advertising expenditure. In their study, Killick and Griffiths (2020) expressed concerns regarding the targeted nature of social media advertising and its potential impact on younger demographics. This is further compounded by the fact that young people, who are in a formative stage of their lives, may be more vulnerable to gambling harm due to their inclination towards risk-taking activities (Duell et al., 2018; Figner & Weber, 2011; Steinberg, 2004; Willoughby et al., 2021; Wilson & Daly, 1985) and their susceptibility to persuasive advertising that may normalise gambling and increase the excitement of sports broadcasts (Deans et al., 2017; McGee, 2020).

As a result of these developments, policymakers are considering implementing measures to ensure that these advertisements do not have a negative impact, particularly on young people and vulnerable groups. The Industry Group for Responsible Gambling (2020) in the UK established a code of conduct that prohibited gambling advertising during sports broadcasts before 9:00 p.m. in an effort to limit the exposure to sports betting advertising to individuals under the age of 18. Importantly, this code does not prohibit advertising during live sporting events and is not mandated by law. In Australia, the Australian Communications and Media Authority (2021) has enforced a ban on sports betting advertising during live sports broadcasts on television and streaming services from 5:00 a.m. to 8:30 p.m. Specifically, gambling advertising is prohibited within 5 min before and after a live sports broadcast. Commentators are also not allowed to promote betting odds within 30 min before and after the live broadcast of the sports event. During the hours of 8.30 p.m. and 5.00 a.m., the rules regarding gambling advertising are more relaxed. Advertising is allowed before and after the event, as well as during scheduled and unscheduled breaks. However, it is not permitted during the event. One evident flaw with the policy is that it does not apply to replays of sporting events or the numerous other media sources that promote sports betting. For example, these rules do not apply to social media advertising, which is where betting companies are most likely to promote betting (National Audit Office, 2020), where people report experiencing “intrusive” exposure to betting advertising (Killick & Griffiths, 2020), and where young people and adolescents consume most of their media (Auxier & Anderson, 2021; Twenge et al., 2019). Other countries have enacted stricter advertising regulations. In 2020, the Spanish government restricted the broadcasting of gambling advertisements on audio-visual media platforms such as television, radio, and YouTube, as well as prohibiting static advertising and sports team sponsorship (Yogonet, 2021). These restrictions confine the broadcasting of gambling advertisements to the hours between 1:00 a.m. and 5:00 a.m. These restrictions were imposed in response to growing concerns about the potential harms associated with gambling and the influence of betting advertisements on vulnerable populations such as young people and problem gamblers.

### The Role of Betting Products and Inducements

An element of sports betting that differentiates it from other common forms of gambling is the ability for sports betting companies to attract customers through a myriad of betting products and inducements. There are numerous forms of wagering products that can be advertised and distinguished depending on the odds, the number of bets that can be placed, the types of outcomes, and the timing of the gambling opportunities. For example, in addition to being able to place bets on the outcomes of matches, people in some jurisdictions can engage in in-play betting. These bets might include micro or exotic bets on particular game or match events (e.g., who scores the next goal). As a result, a bettor can place multiple in-play bets during a match and learn the outcome of those bets seconds after placing them. This shorter interval between placing a bet and learning its outcome and the frequency with which one can place bets mean that in-play betting may be a particularly strong reinforcer of gambling behaviour (Newall et al., 2021). Sports betting companies also offer complex sets such as combination bets that allow people to bet on multiple outcomes (e.g., doubles or triples) or bet on accumulators or multiple bets that involve the multiplication of odds based on the co-occurrence of several outcomes. Studies have indicated that these more unusual types of bets, especially micro-bets, are more popular among high-risk gamblers (Gainsbury et al., 2020; Hing et al., 2017a; Lopez-Gonzalez et al., 2018; Russell et al., 2018). This preference could be attributed to their inclination towards risk-taking behaviour (Ioannidis et al., 2019).

Sports betting frequently includes a variety of incentives that encourage gambling. These can involve bonuses associated with signing up for a new account or service, betting offers (e.g., cash-back offers, increased odds), or special time-limited deals. Marketing research shows that incentives, akin to sign-up bonuses or cash bonuses, lead people to make impulse purchases (Bandyopadhyay et al., 2021). Unsurprisingly, this generalises to gambling, as research has found that betting incentives, such as bonus bets and sign up offers, are linked with the intensification of betting behaviour (Deans et al., 2017; Hing et al., 2014, 2017b; Lopez-Gonzalez et al., 2019). However, not all research necessarily shows that this influence is confined just to higher risk gamblers (e.g., Hing et al., 2018, 2019). These inducements are likely to appeal to young people and may persuade them to increase their sports betting because they are advertised in media that young people consume and frequently include time-limited offers that can cause a fear of missing out (FOMO).

### Harm Mitigation Strategies

Aside from the advertising of gambling products, there are also advertising campaigns for tools or strategies that seek to mitigate gambling-related harm. These tools or messages are commonly known as *responsible gambling tools* or *responsible gambling messages*. However, there is a perception that the phrase *responsible gambling* shifts the responsibility onto the bettor and absolves the betting operator of accountability for providing a potentially harmful product. Therefore, we will adopt the phrase *harm mitigation strategies* when referring to such strategies. Various studies have used data from online betting operators to assess the effectiveness of different approaches to mitigate gambling-related harms, such as voluntary and mandatory deposit limits, pop-up messaging, breaks-in-play, and voluntary exclusion or account closure features. In a matched-pairs study (Auer & Griffiths, 2022), it was found that the provision of personalised behavioural feedback resulted in a reduction in gambling behaviour (as evidenced by the amount and frequency of deposits, the amount wagered, and the time spent gambling). Likewise, Auer and Griffiths (2016) found, using a real-world experimental design, that personalised behaviour feedback decreased wagering behaviour as measured by theoretical loss, profit/loss, and amount wagered. However, two meta-analyses note that only a handful of studies have investigated the efficacy of personalised feedback interventions, and the observed effect among these studies seems to be modest, indicating the necessity for additional research to gain a clearer understanding of their effectiveness (Peter et al., 2019; Saxton et al., 2021). Mandatory breaks in play have also been shown to reduce the intensity of gambling activity. Hopfgartner et al. (2021) found that breaks in play increased the amount of time between gambling sessions while having no rebound effect on gambling behaviour (i.e., an increased intensity of wagering after a forced break in play). On balance, the existing research suggests that both mandatory and voluntary harm mitigation strategies may be effective; however, more research is needed to determine whether betting companies adequately promote these tools, particularly to at-risk gamblers.

### Present Study

In the present study, we aimed to investigate the frequency at which young people recalled coming across sports betting advertisements during a significant sporting event, specifically the 2022 FIFA World Cup. Our first objective was to explore the advertising channels through which young people recalled encountering the most advertising for sports betting. Our second objective was to investigate the bet types and betting inducements that young people recalled encountering the most advertising for and found the most appealing. Our final objective was to investigate how frequently young people recalled coming across advertising for harm mitigation strategies. The young people in this study were between the ages of 18 and 24, which is in line with the United Nations' (1981) definition of young people (Di Censo et al., 2023). Participants were recruited from the United Kingdom, where football (soccer) is the most watched sport. A central part of our analysis was a comparison of higher-risk and lower-risk gamblers based on their Problem Gambling Severity Index (PGSI) scores. We hypothesised that higher-risk gamblers would be more attracted to advertisements for more exotic and complex bet types and betting inducements than lower-risk gamblers.

## Method

### Participants

The Prolific online research panel was used to recruit 190 participants aged between 18 and 24 who lived in the United Kingdom, regularly watched football (soccer), and had watched at least one game from the 2022 FIFA World Cup. Additionally, participants were required to have prior experience with online gambling (i.e., having engaged in online gambling at least once, excluding lotteries). To determine the appropriate sample size, a power analysis was conducted using the G*Power software (Faul et al., 2009). The analysis indicated that a sample of 174 participants would be sufficient to achieve a medium effect size for our main comparison (i.e., the difference in total exposure to sports betting advertising between higher- and lower-risk gamblers).

### Measures

#### Demographics

Demographic information, including age and sex details, were requested. Participants were also asked about their typical involvement in sports betting using an ordinal frequency scale with the following response categories: Never (0), less than once a month (1), once a month (2), 2–3 times a month (3), weekly (4), 2–6 times a week (5), and daily (6). Participants were also asked if they had watched at least one match of the 2022 FIFA World Cup. Those who had not were excluded from the study.

#### PGSI

The Problem Gambling Severity Index (PGSI) was used to determine participants' problem gambling scores (Ferris & Wynne, 2001). This scale has nine items with four possible responses, ranging from 0 (never) to 3 (almost always). The PGSI has a score range of 0 to 27. The scores are usually interpreted into four categories: non-problem (0), low risk (1–2), moderate risk (3–7), and problem gambler (8 +). A median split was used to divide participants into two groups: lower-risk gamblers (non-problem to lower-risk gamblers) and higher-risk gamblers (moderate-risk to problem gamblers). Cronbach's alpha was 0.85, indicating that there was a high level of internal consistency.

#### Exposure to Sports Betting Advertising Scale

Participants were asked how often they had viewed sports betting advertising in the previous week (i.e., during the final week of the 2022 FIFA World Cup) via the following mediums: television, radio, outdoor, social media, email or text messaging, push notifications, internet advertising banners, and during sports commentary. Participants reported their exposure on a 7-point ordinal frequency scale, ranging from 0 viewings in the previous week to 25 + viewings in the previous week. These data were used to calculate a score for “total exposure to advertising during the past week” by adding the scores for each item. This scale has a score range from 0 to 30. Cronbach's alpha for the scale was 0.75, which indicates acceptable internal consistency (Tavakol & Dennick, 2011).

#### Exposure to Advertising for Bet Types and Betting Inducements

We asked participants to indicate whether they recalled encountering advertisements for the any of the bet types and betting inducements presented in Table 1 during the 2022 FIFA World Cup. In addition, we asked participants to identify whether or not any of the advertisements they had observed appealed to them.

**Table 1:** Participants were asked if they encountered advertising for the following bet types and betting inducements

Advertising for bet types	Advertising for betting inducements
Match result	Sign up offers (i.e., bonus bets or bonus cash when signing up to an operator)
Micro-betting or proposition betting (i.e., bets for events within a match such as which player will score a goal, which team will have the most shots on target or which team will have the most offsides)	Deposit match (i.e., deposit matched in bonus bets or bet credits)
In-play betting (i.e., bets made during the match)	Bonus bet (e.g., $10 bonus bet to use on the World Cup)
Multi-bets (i.e., combination of several single bets into one bet with several legs)	Increased odds (enhanced odds, boosted odds, etc.) for single bets
Same game multi-bets	Extra winnings on multi-bets (i.e., % increase in winnings per leg of a multi-bet)
Combo bets (i.e., combination of all possible singles, doubles, trebles, etc., for a number of selections)	Reduced risk (i.e., stake refunded if a leg of multi-bet fails, or a player has been substituted)
Player pick (e.g., Messi or Lewandowski to score)	Extra chance (i.e., if a bet is not settled during normal time, it can be settled during extra time)
Handicap (e.g., Team A + 1.5 goals)	Lengthening odds match (i.e., if odds increase for a bet after the wager was placed, you will be paid the higher odds)
Double chance (e.g., either Team A or Team B to win, either Team A or a draw)	Cash-out (i.e., return a stake in full or partially before the match has finished)
Outright (i.e., who will win the FIFA World Cup)	Popular multi-bets (i.e., operators post multi-bets that are commonly selected by other users or endorsed by the operator)
	Chat rooms (e.g., bet with mates)

#### Advertising for Harm Mitigation Tools

Participants indicated on a dichotomous scale whether they recalled encountering advertisements for harm mitigation tools in the previous month (see Table 2). A variable “total awareness of harm mitigation tools” was scored by adding the participants’ awareness of advertisements for harm mitigation tools.

**Table 2:** Advertising for harm mitigation tools

Pre-commitment (i.e., limits on the amount you can deposit or amount you can lose in a betting session)
Reality checks/check ins (i.e., pop-up message when you have been logged in for a specified amount of time, or deposited, bet or lost a specified amount of money)
Curfew (i.e., self-exclusion from a sports betting account during a specified period of the day)
Self-exclusion (i.e., prohibits you from logging in for a specified period (1 day, 2 weeks, 6 months, 2 years, etc.))
Activity tracking/Play summary
Self-assessments (i.e., assessing if your gambling behaviour is problematic)
Harm mitigation tips

### Procedure

Prolific respondents with the characteristics described above were compensated $24.60 AUD per hour for the survey, with a mean completion time of 5 min and 48 s on average ($2.38 AUD). The main survey was hosted on Qualtrics, where participants completed the demographics, gambling questions, and those relating to their exposure to advertising. The data was quality checked to eliminate any patterned responses, logically inconsistent responses, or very short survey completions. The current study was approved by the Human Research Ethics Subcommittee of the University of Adelaide's School of Psychology; project number: H-2022–22/95.

### Statistical Approach

Several statistical approaches were used to analyse the data for the current paper: a Friedman test to determine whether there were significant differences between the advertising channels through which respondents reported encountering sports betting advertising; Wilcoxon signed-rank tests to identify the advertising channels through which participants recalled more advertising; and Mann–Whitney U tests to compare differences between higher-risk and lower-risk gamblers' median recall to sports betting advertising for each advertising channel. We also determined whether higher-risk gamblers reported more total exposure to sports betting advertising in the previous week than lower-risk gamblers using an independent samples t-test. Due to the highly negatively skewed nature of the data and the interest in comparing people who did and did not meet the standard PGSI criteria for higher-risk gambling, group comparisons were used.

Next, we used chi-squared tests to examine whether higher-risk gamblers were more likely than lower-risk gamblers to recall encountering advertisements for specific bet types and betting inducements and whether they found them more appealing. In a number of categories, the expected cell counts were below five, so we excluded them. Finally, we conducted a Mann–Whitney U test to assess whether higher-risk gamblers were more likely to recall encountering advertisements for harm mitigation tools than lower-risk gamblers.

## Results

### Sample Characteristics

A total of 190 people were recruited to participate in this study. All participants were between the ages of 18 and 24, with an average age of 22. The majority of participants were male (86.8%). The survey contained a question about participants' regular sports betting participation. On average, participants gambled between two and three times per month. Very few respondents (4.2%) reported never having wagered on sports. On the PGSI, participants scored an average of 3.72 (*Md* = 3), indicating that the average participant is a moderate-risk gambler. See Table 3 for further details.

**Table 3:** Description of sample (*N* = 190)

		*n*	%
Age	18	7	3.70
	19	8	4.20
	20	21	11.10
	21	35	18.30
	22	46	24.10
	23	38	19.90
	24	35	18.30
Sex	Male	165	86.80
	Female	24	12.60
Regular sports betting participation	Never	8	4.20
	Less than once a month	43	22.50
	Once a month	28	14.70
	2–3 times a month	38	19.90
	Weekly	37	19.50
	2–6 times a week	29	15.20
	Daily and more often	7	3.70
PGSI standard categories	Non problem	40	20.90
	Low risk	49	25.80
	Moderate risk	75	39.30
	Problem	26	13.60
Total		190	

### In Which Forms of Media Did Young People Recall Encountering the Most Advertising for Sports Betting?

Table 4 summarises the main media locations where young people encountered sports advertising. Participants reported encountering sports betting advertising most frequently on social media (i.e., viewing 10 to 14 sports betting advertisements in the past week), followed by television and internet advertising banners (i.e., viewing 5 to 9 sports betting advertisements in the past week). Young people more frequently reported encountering sports betting advertising through modern media, such as the internet and social media, than through traditional media. A Friedman test found that there were significant differences in recall of advertising through media channels, *χ*2(8) = 578.775, *p* < 0.001. Pairwise comparisons with the Bonferonni correction were conducted to determine if there were significant differences between the three media channels through which young people most often encountered advertising. These comparisons showed that participants were significantly more likely to encounter sports betting advertisements on social media (*Md* = 3) than on television (*Md* = 2) (*p* = 0.002) or internet advertising banners (*Md* = 2) (*p* < 0.001). There were no differences between television and internet advertising banners (*p* = 1.00).

**Table 4:** Frequencies of recalling gambling advertising through various mediums in the past week

Advertising medium		Frequency							Median
		0	1–4	5–9	10–14	15–19	20–24	25 +	
Television	*n*	19	69	44	31	11	6	10	5–9
	%	9.9	36.1	23.0	16.3	5.8	3.1	5.2	
Radio	*n*	142	33	9	4	2	0	0	0
	%	74.3	17.4	4.7	2.1	1.0	0.0	0.0	
Print (newspaper, magazines, etc.)	*n*	143	33	11	2	1	0	0	0
	%	75.3	17.3	5.8	1.0	0.5	0.0	0.0	
Outdoor advertising	*n*	65	78	28	14	5	0	0	1–4
	%	34.0	40.8	14.7	7.3	2.6	0.0	0.0	
Social media feeds	*n*	14	24	46	40	35	9	22	10–14
	%	7.3	12.6	24.1	20.9	18.3	4.7	11.6	
Email or text message advertising	*n*	67	56	28	22	0	12	5	1–4
	%	35.1	29.3	14.7	11.5	0.0	6.3	2.6	
Push notifications	*n*	89	45	32	12	8	1	3	1–4
	%	46.6	23.6	16.8	6.3	4.2	0.5	1.6	
Internet advertising banners	*n*	26	60	40	30	19	9	6	5–9
	%	13.6	31.4	20.9	15.7	9.9	4.7	3.2	
During sports commentary	*n*	80	52	34	11	7	5	1	1–4
	%	41.9	27.2	17.8	5.8	3.7	2.6	0.5	

We then investigated whether higher-risk gamblers recalled encountering sports betting advertising through different media than non-problem gamblers. Since the data were not normally distributed, a series of Mann–Whitney U tests comparing sample medians were used. When compared to lower-risk gamblers, higher-risk gamblers were more likely to recall sports betting advertising via television (*U* = 5508, *z* = 2.774, *p* = 0.006), radio (*U* = 5109.50, *z* = 2.140, *p* = 0.032), social media (*U* = 5739.50, *z* = 3.349, *p* = 0.001), email or text messaging (*U* = 5754.50, *z* = 3.462, *p* = 0.001), push notifications (*U* = 5581.50, *z* = 3.065, *p* = 0.002), internet advertising banners (*U* = 5635, *z* = 3.091, *p* = 0.002), and sports commentary (*U* = 5232, *z* = 2.056, *p* = 0.040). Higher-risk gamblers were not more likely to encounter sports betting advertising via print (*U* = 4533.50, *z* = 0.137, *p* = 0.891) or outdoor advertising (*U* = 4951.50, *z* = 1.283, *p* = 0.200).

### Is There a Difference in the Recall of Sports Betting Advertising Between Higher-Risk and Lower-Risk Gamblers?

We conducted an independent samples t-test to evaluate whether there are differences in total recall of sports betting advertising exposure between higher- and lower-risk gamblers. Higher-risk gamblers (*M* = 14.04, *SD* = 6.32) recalled more sports betting advertising than lower-risk gamblers (*M* = 10.18, *SD* = 6.31), *t*(188) = 4.205, *p* =  < 0.001, *d* = 0.61. Cohen's *d* indicates a moderate effect size.

### Which Bet Types and Inducements Do Young People Recall Encountering the Most Advertisements for?

#### Bet Types

**Fig. 1:**
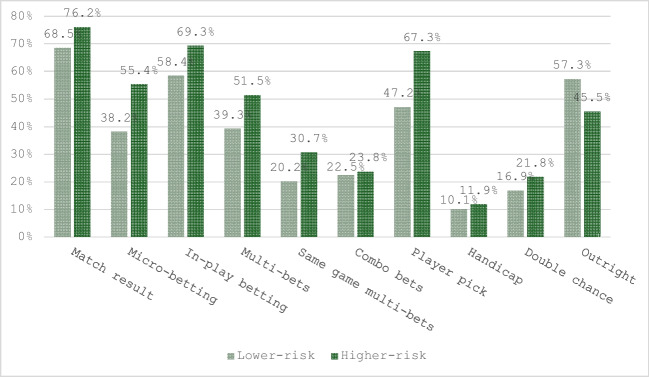
Recall of advertising for bet types during the 2022 FIFA World Cup

Figure 1 depicts the frequency with which participants recalled encountering advertisements for different bet types. Participants generally recalled encountering the most advertising for the bet types “match results,” “in-play betting,” and “player picks” of any bet type. Advertisements for “handicap,” “double chance,” and “combo” bet types were recalled the least. Few participants (4.2%) recalled not encountering advertising for any bet types. We used chi-square tests to determine whether recall of advertising for bet types differed significantly between higher-risk and lower-risk gamblers. Chi-square tests revealed that higher-risk gamblers were more likely to recall encountering advertisements for “micro-betting” and “player picks” than lower-risk gamblers (see Table 5).

**Table 5:** Comparison of higher-risk and lower-risk gamblers' recall of advertising for bet types

	Problem Gambling Severity Index		Total	χ2	*p*
	Lower-risk	Higher-risk			
Match result					
*n* (% within PGSI)	61 (68.50%)	77 (76.20%)	138 (72.60%)	1.410	.235
Micro-betting					
*n* (% within PGSI)	**34 (38.20%)**	**56 (55.40%)**	**90 (47.40%)**	**5.642**	**.018**
In-play betting					
*n* (% within PGSI)	52 (58.40%)	70 (69.30%)	122 (64.20%)	2.437	.119
Multi-bets					
*n* (% within PGSI)	35 (39.30%)	52 (51.50%)	87 (45.80%)	2.818	.093
Same game multi-bets					
*n* (% within PGSI)	18 (20.20%)	31 (30.70%)	49 (25.80%)	2.709	.100
Combo bets					
*n* (% within PGSI)	20 (22.50%)	24 (23.80%)	44 (23.20%)	0.044	.833
Player pick					
*n* (% within PGSI)	**42 (47.20%)**	**68 (67.30%)**	**110 (57.90%)**	**7.869**	**.005**
Handicap					
*n* (% within PGSI)	9 (10.10%)	12 (11.90%)	21 (11.10%)	0.151	.698
Double chance					
*n* (% within PGSI)	15 (16.90%)	22 (21.80%)	37 (19.50%)	0.733	.392
Outright					
*n* (% within PGSI)	51 (57.30%)	46 (45.50%)	97 (51.10%)	2.618	.106
Total	89	101	190		

#### Betting Inducements and Features

**Fig. 2:**
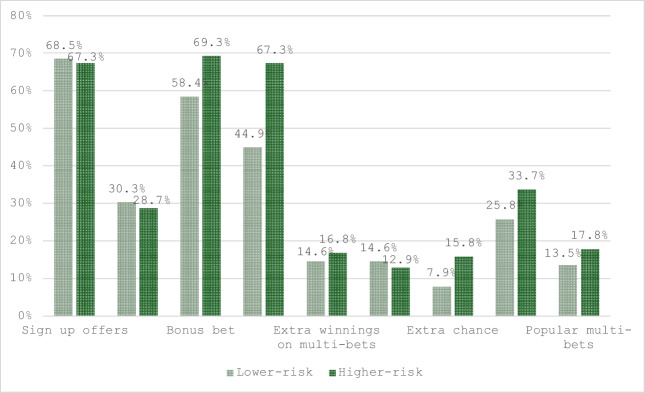
Recall of advertising for betting inducements and features during the 2022 FIFA World Cup

Participants recalled encountering the most advertisements for “sign-up offers,” “bonus bets,” and “increased odds for single bets.” The betting inducements that participants recalled least frequently were those that offered “lengthening odds match,” “reduced risk,” and “extra chances.” Advertisements for features such as “chat rooms” and “popular multi-bets” were rarely recalled. Few participants (10.5%) did not recall any advertisements for betting inducements or features (see Fig. 2). A series of chi-square tests revealed that higher-risk gamblers recalled advertisements for “increased odds” more frequently than lower-risk gamblers (see Table 6).

**Table 6:** Comparison of higher-risk and lower-risk gamblers' recall of advertising for betting inducements and features

	Problem Gambling Severity Index		Total	χ2	*p*
	Lower-risk	Higher-risk			
Sign up offers					
*n* (% within PGSI)	61 (68.5%)	68 (67.3%)	129 (67.9%)	0.032	.858
Deposit match					
*n* (% within PGSI)	27 (30.3%)	29 (28.7%)	56 (29.5%)	0.060	.806
Bonus bet					
*n* (% within PGSI)	52 (58.4%)	70 (69.3%)	122 (64.2%)	2.437	.119
Increased odds					
*n* (% within PGSI)	**40 (44.9%)**	**68 (67.3%)**	**108 (56.8%)**	**9.662**	**.002**
Extra winnings on multi-bets					
*n* (% within PGSI)	13 (14.6%)	17 (16.8%)	30 (15.8%)	.176	.675
Reduced risk					
*n* (% within PGSI)	13 (14.6%)	13 (12.9%)	26 (13.7%)	.121	.728
Extra chance					
*n* (% within PGSI)	7 (7.90%)	16 (15.8%)	23 (12.1%)	2.829	.098
Cash-out					
*n* (% within PGSI)	23 (25.8%)	34 (33.7%)	57 (30.0%)	1.378	.240
Popular multi-bets					
*n* (% within PGSI)	12 (13.5%)	18 (17.8%)	30 (15.8%)	.670	.413
Total	89	101	190		

Lengthening odds match and chat rooms were removed because they failed to reach the minimum expected cell count of five

### Advertisements for Bet Types or Betting Inducements That Are Appealing to Higher-Risk Gamblers

Analyses were conducted to determine which advertisements for bet types and betting inducements appealed most to higher-risk gamblers. Only those who recalled encountering the advertisements provided data. Chi-square tests revealed that advertisements for “match result” and “bonus bets” were more appealing to higher-risk gamblers than to lower-risk gamblers (see Table 7).

**Table 7:** Comparison of the appeal of advertising for bet types and betting inducements between problem gambling categories

	Problem Gambling Severity Index		Total	χ2	*p*
	Lower-risk	Higher-risk			
Match result					
*n* (% within PGSI)	**25 (41%)**	**47 (61%)**	**72 (52.2%)**	**5.486**	**.019**
Micro-betting					
*n* (% within PGSI)	13 (38.2%)	31 (55.4%)	44 (48.9%)	2.482	.115
In-play betting					
*n* (% within PGSI)	18 (34.6%)	26 (37.1%)	44 (36.1%)	0.083	.774
Multi-bets					
*n* (% within PGSI)	13 (37.1%)	25 (48.1%)	38 (43.7%)	1.017	.313
Same game multi-bets					
*n* (% within PGSI)	4 (21.1%)	15 (48.4%)	19 (38.8%)	3.284	.070
Combo bets					
*n* (% within PGSI)	6 (30%)	13 (54.2%)	19 (42.2%)	2.597	.107
Player pick					
*n* (% within PGSI)	17 (40.5%)	31 (45.6%)	48 (43.6%)	0.276	.599
Double chance					
*n* (% within PGSI)	6 (40%)	7 (31.8%)	13 (35.1%)	0.262	.609
Outright					
*n* (% within PGSI)	20 (39.2%)	26 (56.5%)	46 (47.4%)	2.905	.088
Sign up offers					
*n* (% within PGSI)	26 (42.6%)	35 (51.5%)	61 (47.3%)	1.010	.315
Deposit match					
*n* (% within PGSI)	8 (29.6%)	13 (44.8%)	21 (37.5%)	1.378	.240
Bonus bet					
*n* (% within PGSI)	**21 (40.4%)**	**48 (68.6%)**	**69 (56.6%)**	**9.648**	**.002**
Increased odds					
*n* (% within PGSI)	17 (42.5%)	38 (55.9%)	55 (50.9%)	1.805	.179
Cash-out					
*n* (% within PGSI)	10 (43.5%)	11 (32.4%)	21 (36.8%)	0.730	.393

Handicap, extra winnings on multi-bets, reduced risk, extra chance Lengthening odds match, popular multi-bets and chat rooms were removed because they failed to reach the minimum expected cell count of five

### Recall of Advertising for Harm Mitigation Tools

We also examined young people's recall of advertising for harm mitigation tools (see Table 8). Over half of participants (53.7%) did not recall having encountered any advertising for harm mitigation tools, in contrast to the higher percentages of 95.8% and 89.5% who reported encountering at least one advertisement for bet types and betting inducements, respectively. Among the advertisements that participants reported encountering, those promoting reality checks (26.8%) and harm mitigation tips (23.2%) were the most frequently reported. We conducted further statistical analysis using a Mann–Whitney U test, which revealed that higher-risk gamblers (*Md* = 1) were significantly more likely to have encountered advertisements for harm mitigation tools compared to lower-risk gamblers (*Md* = 0); *U* = 5191.50, *z* = 2.018, *p* = 0.044.

**Table 8:** Recall of advertising for harm mitigation tools during 2022 FIFA World Cup

Harm mitigation tools	*n* (%)
No	102 (53.7%)
Pre-commitment	26 (13.7%)
Reality checks/check ins	51 (26.8%)
Curfew	9 (4.7%)
Self-exclusion	23 (12.1%)
Activity tracking/Play summary	15 (7.9%)
Self-assessments	9 (4.7%)
Harm mitigation tips	44 (23.2%)
Total	279

## Discussion

In this study, we examined young people's recall of sports betting advertising during the 2022 FIFA World Cup as well as the types of advertising they found most appealing. We found that young people frequently encountered sports betting advertisements during the World Cup, with higher-risk gamblers being the most likely to encounter them. Young people were most likely to encounter sports betting advertising through modern media channels such as the internet and social media, with higher-risk gamblers recalling more advertising via personalised media sources. Young people recalled encountering advertisements for risky bet types and promotions such as in-play betting, sign-up offers, bonus bets, and increased odds on a regular basis. Higher-risk gamblers were more likely than lower-risk gamblers to recall advertisements for micro-bets and increased odds. Although the majority of young people were unaware of advertising for harm mitigation tools, higher-risk gamblers were more aware of such advertisements than lower-risk gamblers. In general, higher-risk gamblers recalled more sports betting advertising through various media channels, and they were more likely to encounter advertisements for risky bet types and betting inducements than lower-risk gamblers.

### Where Young People Recall Encountering Sports Betting Advertising

This study found that young people, and especially higher-risk gamblers, frequently recalled sports betting advertisements. They recalled advertising most often on social media, followed by television and internet advertising banners. However, they were less likely to recall advertising on traditional media sources such as radio and print. The present study's findings differ from previous studies conducted on larger adult cohorts (e.g., Syvertsen et al., 2021); however, they align with existing knowledge on the media consumption patterns of young people, which indicate that they have a preference for digital media over traditional media (Auxier & Anderson, 2021; Twenge et al., 2019). Higher-risk gamblers recalled more sports betting advertising overall compared to lower-risk gamblers and typically reported encountering more advertising on media channels where they received personalised advertising (e.g., push notifications, emails and text messages). In addition, they were more likely recall advertisements on social media and internet advertising banners, which are usually personalised based on data gathered by third-party web tracking services about a person’s internet activity (Boerman et al., 2017; Mayer & Mitchell, 2012). These findings are consistent with previous research demonstrating that higher-risk gamblers encounter more personalised and direct advertising than their lower-risk counterparts (Syvertsen et al., 2021). Researchers have expressed concerns about digital media advertising, particularly social media advertising (Killick & Griffiths, 2020), and our findings provide additional evidence to support these concerns. Given that young people and high-risk gamblers are frequently exposed to sports betting advertisements online, policymakers should consider advertising restrictions that include these channels, as several European Union countries have done (e.g., Spain, Belgium).

### Advertising for Betting Types

The results of our study indicate that young people frequently recall advertisements promoting player picks, multibets, and in-play betting. In addition, higher-risk gamblers reported encountering micro-betting advertisements more frequently than lower-risk gamblers. These types of bets have been recognised as contributing to harm. For example, multibets were found to be particularly salient, and this could be due to their larger payouts (Hing et al., 2022), which may be appealing to young people. However, the risk that multibets carry over conventional bet types is that all parts of the bet must be successful for the gambler to win the bet, making them less likely to be successful (Newall et al., 2021). Advertising for in-play betting was also frequently reported. Since there is a shorter interval between placing a wager and observing the outcome, in-play betting may be a particularly strong reinforcer of gambling behaviour (Newall et al., 2021) and has been linked with gambling problems (Gainsbury et al., 2020; Hing et al., 2017a; Killick & Griffiths, 2018; Lopez-Gonzalez et al., 2018; Russell et al., 2018). Micro-betting, a form of in-play betting, was found to be particularly salient to higher-risk participants in the present study and has also been found to be associated with problem gambling (Russell et al., 2018). Less frequently observed was advertising for less risky bet types that offered safer betting options (e.g., handicaps and double chance). This may be because sports betting companies advertise them less often because they are less profitable. Young people, who tend to score higher on measures related to risky recreational and financial decision making (Rolison et al., 2014; Steinberg, 2004), may be more interested in riskier bet types with higher payouts and hence pay more attention to their advertising. On balance, this study found that young people were more likely to recall encountering advertisements for riskier bet types that have been associated with problem gambling.

### Advertising for Betting Inducements and Features

The present study revealed that young people most often recalled advertisements for sign-up offers, bonus bets, and increased odds offers. In particular, higher-risk gamblers recalled advertisements for increased odds more frequently and found bonus bet promotions more appealing. Sports betting companies may promote increased odds as a strategy to encourage people to place riskier wagers by enticing them with higher payouts. Killick and Griffiths (2020) found that gamblers were particularly attracted to increased odds inducements due to their erroneous belief that such inducements increased their likelihood of winning a bet. Moreover, higher-risk gamblers, who are known to have a greater sensitivity to reward (Jiménez-Murcia et al., 2016; Wardell et al., 2015), may perceive increased payouts as especially enticing. Notably, only bonus bet promotions were found to be more appealing to higher-risk gamblers than to lower-risk gamblers. Sports betting companies regularly offer bonus bets as a means of incentivising customers’ continued patronage or upon opening a new betting account. In addition, our research indicates that sign-up offers were the most commonly encountered form of betting inducement among young people. Sign-up offers are a common marketing tactic that uses financial incentives to draw in new clients, which can encourage impulsive decision-making (Bandyopadhyay et al., 2021). Previous research has demonstrated that the free bets that are provided by sign-up offers and bonus bets have the potential to intensify gambling behaviour in both problem gamblers (Deans et al., 2017; Hing et al., 2014; Lopez-Gonzalez et al., 2019) and non-problem gamblers (Hing et al., 2018, 2019). The present study illustrates that the betting incentives that previous research has associated with gambling-related harm are typically those for which young people encounter the most advertising.

### Harm Mitigation Tools

The present study revealed that less than half (46.3%) of young people reported encountering at least one advertisement for harm mitigation tools. This was in contrast to the greater proportions of 95.8% and 89.5% who reported encountering at least one advertisement for bet types and betting inducements, respectively, which suggests that harm mitigation tools may not be adequately advertised. The harm mitigation tools that young people encountered the most advertising for were reality checks and harm mitigation tips. The present study did not evaluate whether advertising encouraged young people to use these harm mitigation tools or whether they were effective. While there is preliminary evidence supporting the effectiveness of certain harm mitigation strategies (e.g., Auer & Griffiths, 2016, 2022; Hopfgartner et al., 2021), more research is needed to validate these findings (Harris & Griffiths, 2017; Monaghan & Blaszczynski, 2009; Peter et al., 2019; Saxton et al., 2021). Although the overall efficacy of these harm mitigation resources is still being assessed, it may be advantageous to promote them more widely among youth in order to inform them about the various options available to minimise gambling-related harm.

### Strengths and Weaknesses

The participants provided a retrospective account of sports betting advertisements they encountered in the previous week during the 2022 FIFA World Cup. The brevity of this reporting period may have been beneficial, as it potentially enhanced recall accuracy in comparison to a longer reporting period (e.g., one month). One limitation of this study is that the data collected during a major sporting event may not accurately reflect young people's usual exposure to advertising. Moreover, it is important to note that the data used in this study were collected from the United Kingdom, where sports betting advertising laws are more lenient. Thus, the findings may not generalise to nations where sports betting advertising is subject to more stringent regulations. Another limitation of this study is that it relied on participants’ recall of sports betting advertisements, which is susceptible to recall bias. More involved gamblers, for example, tend to be more attentive to advertising and receive more personalised advertising. Consequently, it is not appropriate to draw conclusions regarding whether exposure to gambling advertising leads to higher problem gambling scores. Finally, the study recruited participants from an online research panel, which could introduce bias as online research panel users may not fully represent the general population of young people.

## Conclusion

The current study revealed that young, higher-risk gamblers are more likely to recall encountering sports betting advertisements primarily through personalised digital media sources. A sizeable proportion of youth recalled encountering advertisements for bet types and betting inducements that are considered harmful, while they are comparatively less likely to recall encountering advertisements for less risky bet types. In addition, this study revealed that young people demonstrate a lower recall of advertising for harm mitigation tools. These findings suggest that there is a need for regulatory interventions that limit the exposure of young people to betting promotions that may contribute to gambling harm. These include the bet types and betting promotions that young people frequently encounter being advertised, including micro-betting, in-play betting, multi-bets, bonus bets, increased odds offers, and sign-up offers. Future research will need to further examine whether different bet types and promotions influence the gambling behaviour of young individuals and whether they contribute to an increase in gambling harm.

## Data Availability

Data supporting the findings of the present study are available upon reasonable request from the corresponding author.
